# Overexpressed MPS-1 contributes to endometrioma development through the NF-κB signaling pathway

**DOI:** 10.1186/s12958-021-00796-z

**Published:** 2021-07-15

**Authors:** Yang Liu, Junyan Ma, Liqi Zhang, Jun Lin, Xiaohua Liu

**Affiliations:** 1 Department of Obstetrics and Gynecology, Shanghai First Maternity and Infant Hospital, School of Medicine, Tongji University, Shanghai, 200092 People’s Republic of China; 2 Department of Key Laboratory, Women’s Hospital, School of Medicine, Zhejiang University, Hangzhou, 310006 People’s Republic of China; 3 Department of Obstetrics and Gynecology, Women’s Hospital, School of Medicine, Zhejiang University, Hangzhou, 310006 People’s Republic of China

**Keywords:** Endometrioma, Metallopanstimulin-1 (MPS-1), NF-κB signaling pathway, Ectopic endometrial stromal cells

## Abstract

**Background:**

Endometriosis is a benign gynecological disease that shares some characteristics with malignant tumors and affects approximately 10% of women of reproductive age. Endometrioma refers to endometriosis that appears in the ovary. Metallopanstimulin-1 (MPS-1) is a component of the 40S subunit of ribosomes that has extra-ribosomal functions that contribute to the development of diseases. This study aimed to explore the expression pattern and role of MPS-1 in endometrioma development.

**Methods:**

Quantitative real time polymerase chain reaction, western blotting, immunohistochemistry, and enzyme-linked immunosorbent assay were used to determine the expression of MPS-1 in patients with endometrioma. Following the successful knockdown of MPS-1 by siRNA, CCK-8 assays, flow cytometry, and transwell assays were performed to detect ectopic endometrial stromal cells (EcESCs) proliferation, the rate of apoptosis, and cell cycle, migration, and invasion, respectively. Western blotting was used to explore the effect of MPS-1 knockdown on protein levels in the NF-κB signaling pathway.

**Results:**

The expression of MPS-1 was significantly higher in endometrioma and the serum of endometrioma patients than in the patients without endometriosis. In addition, the downregulation of MPS-1 expression inhibited EcESCs proliferation, migration, and invasion. This downregulation led to the arrest of the EcESCs cycle in the G0/G1 phase and apoptosis and depressed the NF-κB signaling pathway.

**Conclusion:**

MPS-1 can regulate EcESCs proliferation, motility, invasion, apoptosis, and cell cycle via the NF-κB signaling pathway in endometrioma. This may contribute to the formation or development of endometriotic foci. This study suggests the potential role of MPS-1 in the pathogenesis of endometriosis and enabled further research into the use of MPS-1 in the clinical diagnosis of endometrioma.

**Supplementary Information:**

The online version contains supplementary material available at 10.1186/s12958-021-00796-z.

## Introduction

Endometriosis, the presence of endometrial-like tissues outside the uterus cavity and myometrium, is a benign gynecological disease with characteristics resembling malignant tumors [1]. It affects approximately 10% of women of reproductive age, and up to 50% of endometriosis patients are infertile [2]. Endometrioma refers to endometriosis that appears in the ovary, taking up to 44% of endometriosis patients. Approximately 1% of endometriosis cases develop into ovarian carcinoma [3]. Although research on endometriosis has been ongoing for decades, the precise pathogenesis remains inconclusive. Recent sequencing data show that aberrant gene expression contributes to the pathogenesis of endometriosis [4–6].

Metallopanstimulin-1 (MPS-1), also called RPS27, is a component of the 40S subunit of ribosomes [7]. MPS-1 is overexpressed in various tissues in the proliferative phase in both benign and malignant tissues [7–9]. MPS-1 is secreted into the extracellular space and can be used to detect neoplastic diseases in their early stage or used for monitoring disease progression during follow-up [10]. As a ribosomal protein, MPS-1 can participate in ribosomal biogenesis; notably, it can affect other biological functions by regulating gene translation, transcription, and DNA repair [11]. For example, MPS-1 can bind to MDM2. This protects p53 from MDM2-mediated ubiquitination and degradation, thus modulating p53 transcriptional activity [7]. In gastric cancer, MPS-1 contributes to carcinogenesis and predicts poor outcomes as it regulates cell proliferation and apoptosis through NF-κB signaling [12]. NF-κB is a critical transcriptional factor that is inactive when bound to its specific inhibitor IκB [13]. In the canonical activation of NF-κB, IκB kinase induces IκB degradation through phosphorylation and polyubiquitination, resulting in the release and activation of NF-κB [14]. Activated NF-κB then translocates to the nucleus and regulates the expression of cytokines or behavior-related genes. NF-κB signaling can affect angiogenesis, inflammation, invasion, and oxidative stress in endometriosis [15, 16]. Progesterone and GnRH agonists are used as treatments for endometriosis as they alleviate symptoms and inhibit the NF-κB signaling pathway [17, 18]. Vascular cell adhesion molecule-1 (VCAM-1), a downstream target of NF-κB signaling, is overexpressed in ectopic lesions and the serum of endometriosis patients [19]. VCAM-1 not only mediates the functions of endometriotic cyst stromal cells [20] but also participates in the immune response of the endometriotic microenvironment [21].

Considering that in endometriosis the endometrium grows in ectopic sites abnormally and possesses malignant biological characteristics, this study aimed to explore the functions and role of MPS-1 in endometrioma. To the best of our knowledge, this is the first study to report the differential expression and role of MPS-1 in endometriosis development.

## Materials and methods

### Clinical specimen collection

The study was approved by the Ethics Committee of Women’s Hospital, School of Medicine, Zhejiang University (ethics number: 20190037). All the participants signed the informed consent. Ectopic tissues and paired eutopic endometrium were collected from 20 patients who were diagnosed with III/IV stage endometriosis with endometrioma according to the revised American Fertility Society (rAFS) scoring system. All patients underwent hysteroscopy combined with laparoscopy and histopathological determination as they either experienced persistent symptoms and/or developed large cysts. Control endometrium was obtained from 20 non-endometriosis patients who were diagnosed with leiomyoma, uterus septum, or infertility. These patients were confirmed not to suffer from endometriosis using laparoscopy and ultrasound. The 40 patients (proliferative phase) enrolled in the study in 2019 were aged between 18 and 45 years and excluded adenomyosis or malignant diseases and had not received hormonal treatment in the preceding three months.

Serum samples were collected from a separate cohort of 40 patients, and the inclusion and exclusion criteria were consistent with the criteria set above. The clinical information is summarized in Supplementary Table. To avoid repeated freeze–thaw cycles leading to sample degradation, both tissue and serum samples were aliquoted and stored at -80 °C immediately after collection.

### Isolation and culture of ectopic endometrial stromal cells (EcESCs)

Endometrioma were isolated from endometriosis patients in the proliferative phase and aged between 20–35 years old who underwent hysteroscopy combined with laparoscopy. Once lesions were isolated from patients, they were transferred to the laboratory at 4 °C and processed within 1.5 h. The procedures were performed as previously described [22]. EcESCs used in this study were stained for cytokeratin-7 and vimentin and had a passage number of 5 or less. The characterization image of EcESCs was seen in Supplementary Figure.

Quantitative real time polymerase chain reaction (qRT-PCR).

Total RNA was extracted using TRIzol reagent (Invitrogen, USA), and the quality and concentration was evaluated using a Nanodrop spectrophotometer (Thermo Fisher, USA). RNA (1000 ng) was reverse transcribed to cDNA using the PrimeScript™ RT reagent Kit with gDNA Eraser (Takara, Japan). cDNA was amplified using the Applied Biosystems ViiA™ 7 system (ABI, USA) using SYBR® Premix Ex TaqTM kit (Takara, Japan) and appropriate primers (Generay, China). All procedures were performed in accordance with the manufacturer’s instructions. The sequences of the primers used were: MPS-1-forward, 5’-ATGCCTCTCGCAAAGGATCTC-3’, MPS-1-reverse, 5’-TGAAGTAGGAATTGGGGCTCT-3, β-actin-forward, 5’- CATGTACGTTGCTATCCAGGC-3, β-actin-reverse, 5’- CTCCTTAATGTCACGCACGAT-3’. β-actin was used as the reference gene, and fold change was calculated using the 2^−ΔΔCt^ method in order to compare the differential expression of target genes.

### Western blotting

Total protein was extracted using the Minute™ kit (Invent, USA). Western blotting assays were performed according to the manufacturer’s instructions using SurePage™ precast gels (Genscript, China). The antibodies used were anti-GAPDH (ab8245, dilution 1:1000, mouse; Abcam, USA), anti-MPS-1 (ab4385, dilution 1:1000, goat; Abcam, USA), anti-NF-κB p65 (#8242, dilution 1:1000, rabbit; CST, USA), anti-phospho-NF-κB p65 (#3033, dilution 1:1000, rabbit; CST, USA), and VCAM-1 (ab134047, dilution 1:2000, rabbit; Abcam, USA).

Band signals were detected using ImageQuant LAS 4000 (GE Healthcare, Sweden) using the Enhanced ECL Chemiluminescent Substrate Kit (Yeasen, China). Densitometry analysis was performed using Image J software and normalized to GAPDH.

### Immunohistochemistry

Endometrioma and endometrium were formalin-fixed, paraffin-embedded, and cut into 5-μm thick slices. The slices were treated with xylene and a graded series of alcohol for dewaxing and dehydration, followed by antigen repair with sodium citrate buffer and microwave heating. After being equilibrated to room temperature and washed with PBS, the slices were blocked with 10% goat serum at 37 °C for 20 min, and incubated with anti-MPS-1 (ab197382, dilution 1:50, rabbit; Abcam, USA) at 4 °C overnight. After washing three times and incubating with goat anti-rabbit IgG (ab205718, dilution 1:10,000; Abcam, USA) at 37 °C for 1 h, the slices were visualized under the microscope using diaminobenzidine. Subsequently, water flushing, hematoxylin staining, hydrochloric acid alcohol differentiation, dehydration, transparency, and sealing were performed. Samples without a primary antibody were used as negative controls. The slices were photographed using a digital slice scanner. Two pathologists observed and assigned scores based on the area and degree of immunostaining. Final scores were evaluated using the immunoreactive score (IRS) system.

### Enzyme-linked immunosorbent assay (ELISA)

To detect the protein levels of MPS-1 in serum samples, ELISA was performed using the Human 40S ribosomal protein S27 (RPS27) ELISA Kit (CSB-EL020419HU, CUSABIO, China). Frozen serum samples were thawed on ice. Reactions were carried out in accordance with the manufacturer’s instructions.

### Cell transfection

EcESCs at a density of 1 × 10^5^ cells were seeded onto six-well plates. The next day, the EcESCs were transfected with small interfering RNA (siRNA) against MPS-1 (si-MPS-1) or negative control (NC) at a concentration of 40 nmol/L using Lipofectamine RNAiMAX (Invitrogen, USA). siRNAs were synthesized by Genepharma Corporation (Shanghai, China), and the sequences were as follows:

si-MPS-1-sense, 5’-CGCAAAGGAUCUCCUUCAUTT-3’;

si-MPS-1-antisense, 5’- AUGAAGGAGAUCCUUUGCGTT-3’.

si-Negative Control-sense, 5’-UUCUCCGAACGUGUCACGUTT-3’;

si-Negative Control-antisense, 5’-ACGUGACACGUUCGGAGAATT-3’.

### Cell proliferation

EcESCs were seeded onto 96-well plates at a density of 4000 cells/well in 100 μL complete medium. The next day, EcESCs were transfected with siRNAs. At 0, 24, 48, 72, and 96 h post-transfection, 100 μL of culture medium containing 10 μL CCK-8 (Dojindo, Japan) was added into each well, and the plates were incubated at 37 °C for 2 h. Cell proliferation was determined by measuring the absorbance at 450 nm using a Thermo Scientific Microplate Reader (Thermo Fisher, USA).

### Flow cytometry

Flow cytometry was used to detect cell cycle progression and apoptosis. EcESCs were harvested 72 h post-transfection and analyzed using the Annexin V-APC/7-AAD apoptosis kit (MultiSciences, China). Annexin V single-positive EcESCs were classified as early apoptotic cells, and the double-positive EcESCs were classified as late apoptotic and necrotic cells. Cell cycle stages were analyzed by flow cytometry using the Cell Cycle Staining Kit (MultiSciences, China).

### Transwell assays

After 48 h of transfection, 5 × 10^4^ EcESCs in 200 μL DMEM/F12 were seeded onto the upper transwell chamber (BD Falcon, USA). DMEM/F12 (500 μL) with 10% fetal bovine serum serving as the chemoattractant was added to the lower chamber. After 24 h, cells on the upper layer were removed, and EcESCs that migrated to the lower chamber were fixed with methanol, stained with 0.1% crystal violet, and washed with PBS. Using a light microscope at 200X magnification, dried chambers were observed and photographed (Olympus, Japan), and five visual fields were selected randomly for counting.

Transwell assays were also used to detect the invasive ability of EcESCs. Diluted Matrigel (BD Biosciences, USA) was placed on the upper chamber prior to seeding of 1 × 10^5^ transfected EcESCs. After 48 h, cells in the lower chamber were treated and counted as described above.

### Statistical analysis

All experiments were carried out at least three times, and the results are presented as mean ± SEM. Data were analyzed using GraphPad Prism 5 software. The Mann–Whitney test, unpaired Student’s t-test, or paired Student’s t-test were used to compare the differences. Two-tailed tests with *p* < 0.05 were considered as statistically significant.

## Results

### MPS-1 is overexpressed in endometriosis patients

**Fig. 1:**
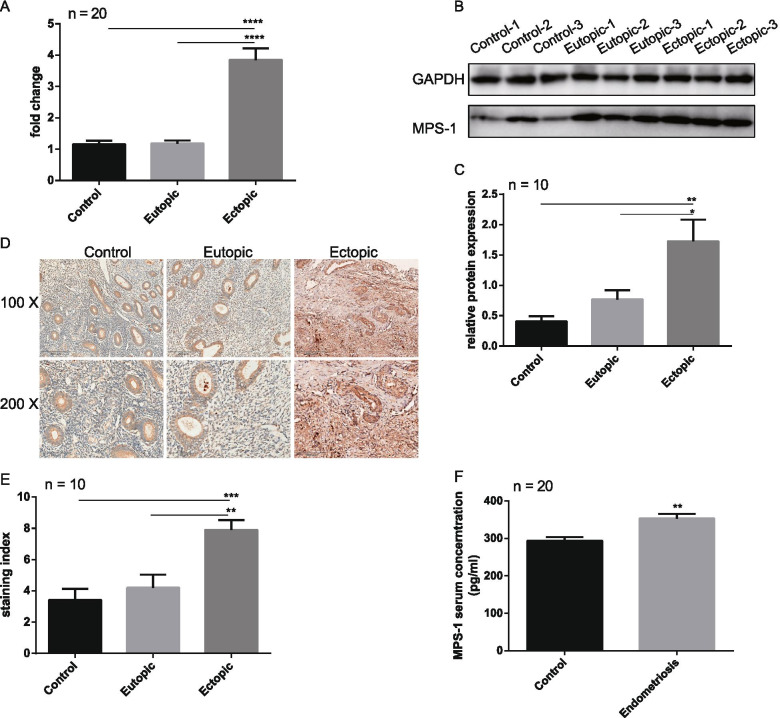
Expression of MPS-1 in endometrium and serum from patients with endometrioma or not. **A** mRNA levels of MPS-1 were evaluated by qRT-PCR in control endometrium (n = 20), eutopic endometrium (n = 20) and ectopic endometrial tissues (n = 20) from patients with endometrioma or not. **B** A representative western blotting image of MPS-1, endometrium and ectopic lesions were from three control patients and three patients with endometrioma. **C** Protein levels of MPS-1 in control endometrium (n = 10), eutopic endometrium (n = 10) and ectopic endometrial tissues (n = 10) were detected by western blotting, evaluated by Image J software, and normalized to GAPDH. **D** Representative images of immunohistochemistry for MPS-1. The magnification is 100 × and 200 × . The scale bar is 200 μm and 100 μm. **E** The immunohistochemistry intensity for MPS-1 in control endometrium (n = 10), eutopic endometrium (n = 10) and ectopic endometrial tissues (n = 10) was evaluated by IRS. **F** Serum MPS-1 levels in control group (n = 20) and endometriosis group (n = 20) were detected by ELISA. Data are presented as mean ± SEM (*p < 0.05; **p < 0.01; ***p < 0.001; ****p < 0.0001)

qRT-PCR results showed that the mRNA levels of MPS-1 were significantly higher in endometrioma compared to both eutopic endometrium and control endometrium. No significant difference in MPS-1 mRNA levels was seen between eutopic and control endometrium (Fig. 1.A). Similarly, the protein levels of MPS-1, as detected by western blotting, were consistent with the qRT-PCR results (Fig. 1.B, C). In addition, immunohistochemistry results showed that MPS-1 express in both endometrial epithelial and stromal cells, and the staining intensity of MPS-1 in endometrioma was strongly positive, while it was weakly positive in the eutopic and control endometrium (Fig. 1.D, E). Using ELISA, we found that serum MPS-1 level was elevated in endometrioma patients compared to patients without endometriosis (Fig. 1.F). Using the median value of serum MPS-1 as a cutoff (314.81 pg/mL), all patients were divided into two groups, namely the low MPS-1 group (concentration < 314.81 pg/mL) and high MPS-1 group (concentration ≥ 314.81 pg/mL) (Table 1). Fifteen of the twenty patients with endometriosis had high concentrations of MPS-1 (*p* < 0.01), while MPS-1 levels showed no difference in patients with leiomyoma, uterus septum, or infertility. Moreover, serum levels of CA-125 were higher in the high MPS-1 group, indicating a positive correlation between CA-125 and MPS-1. Taken together, MPS-1 is overexpressed in the endometriotic tissues and serum of patients with endometrioma.

**Table 1:** The relationship between MPS-1 expression in serum and clinical information

	Low MPS-1 (Conc. < 314.81 pg/ml)	High MPS-1 (Conc. ≥ 314.81 pg/ml)	*p* value
Total patients	20	20	1.000
Endometriosis	5	15	**0.002**^**a**^
Leiomyoma	7	2	0.058
Mediastinal uterus	6	3	0.256
Infertility	3	6	0.256
Age (years)	32.3 ± 8.8	34.8 ± 6.7	0.436
CA-125 (U/ml)	20.97 ± 10.91	38.27 ± 31.52	**0.034**^**a**^

***p***^**a**^ indicated difference with statistical significance

Chi-square tests were used to evaluate categorical variables including total patients, endometriosis, leiomyoma, mediastinal uterus and infertility

Mann–Whitney U tests were used to evaluate the levels of age and CA-125

### MPS-1 downregulation inhibits multiple functions of EcESCs

**Fig. 2:**
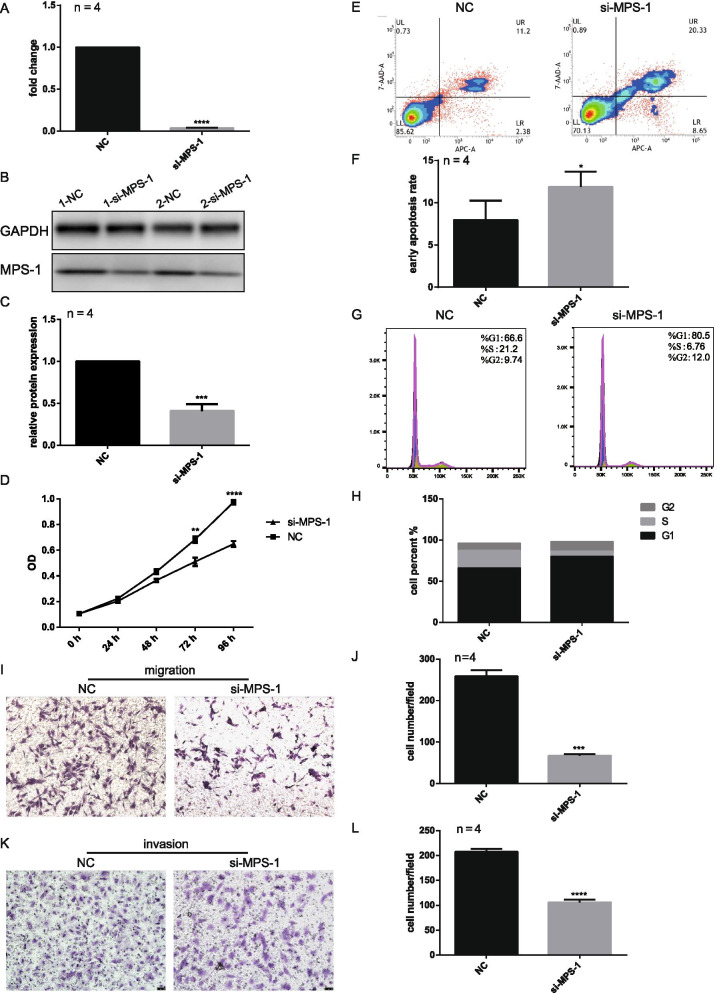
MPS-1 regulates proliferation, migration, invasion, apoptosis and cell cycle of EcESCs. **A** Transfection efficiency of MPS-1 siRNA was detected by qRT-PCR. **B** A representative western blotting image of MPS-1 after EcESCs were transfected with MPS-1 siRNA for 72 h (n = 2). **C** Based on the results of western blotting, knockdown efficiency of MPS-1 siRNA was analyzed by Image J software. **D** The effects of MPS-1 siRNA on proliferation were evaluated by CCK-8 assay. **E**, **G** The representative images of EcESCs apoptosis and cell cycle were depicted by flow cytometry after MPS-1 siRNA transfection for 72 h. **F**, **H** The apoptosis analysis and cell cycle analysis were based on the results of flow cytometry. **I**, **K** Transwell assays were used to determine the effects of MPS-1 siRNA on migration and invasion. The scale bar is 50 μm. **J**, **L** Cell count analysis of Transwell migration and invasion. NC, si-MPS-1 EcESCs transfected with negative control siRNA or MPS-1 siRNA (n = 4). Data are presented as mean ± SEM (*p < 0.05; **p < 0.01; ***p < 0.001; ****p < 0.0001)

To explore the role of MPS-1 in endometriosis, we performed multiple functional assays to detect EcESCs proliferation, migration, invasion, apoptosis, and cell cycle following MPS-1 downregulation. As demonstrated by qRT-PCR and Western blotting, MPS-1 siRNA could significantly inhibit the expression of MPS-1 (Fig. 2.A-C). According to the results of the CCK-8 assays, MPS-1 downregulation inhibited EcESCs proliferation, with a significant difference observed 72 h post-transfection (Fig. 2.D). Additionally, MPS-1 downregulation not only promoted EcESCs apoptosis (Fig. 2.E, F) but also induced EcESCs cycle arrest in the G0/G1 phase (Fig. 2.G, H). Moreover, the transwell migration assay demonstrated that MPS-1 downregulation weakened the migration (Fig. 2.I, J) and invasion ability of EcESCs compared with the NC group (Fig. 2.K, L). These results suggest that MPS-1 dysregulation in EcESCs plays a role in multiple biological processes involved in the progression of endometrioma, including cellular proliferation, cell cycle progression, apoptosis, cell migration and invasion.

### MPS-1 downregulation regulates the NF-κB signaling pathway

**Fig. 3:**
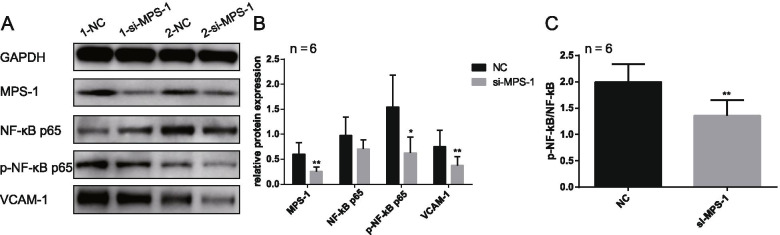
MPS-1 regulates the NF-ĸB signaling pathway in endometrioma. **A** The representative western blotting images of MPS-1, NF-ĸB p65, p-NF-ĸB p65 and VCAM-1 after EcESCs were transfected with MPS-1 siRNA for 72 h (n = 2). **B** The protein levels of MPS-1, NF-ĸB p65, p-NF-ĸB p65 and VCAM-1 were analyzed by Image J software (n = 6). The protein levels were normalized to GAPDH. **C** The changes in phosphorylation levels of NF-ĸB signaling were evaluated by p-NF-ĸB/ NF-ĸB (n = 6). Data are presented as mean ± SEM (*p < 0.05; **p < 0.01)

To investigate whether MPS-1 regulates EcESCs through the NF-κB signaling pathway, protein levels of MPS-1, NF-κB p65, p-NF-κB p65, and VCAM-1 were measured by western blotting following MPS-1 knockdown. As shown in Fig. 3, the protein levels of p-NF-κB p65 and VCAM-1 decreased significantly following MPS-1 knockdown (Fig. 3.A and B). Interestingly, although the changes in NF-κB p65 levels were inconsistent after MPS-1 downregulation (Fig. 3.A), the ratio of p-NF-κB p65 to NF-κB p65 remained low (Fig. 3.C). The results indicate that the NF-κB signaling pathway mediates the regulation of MPS-1 in endometriosis.

## Discussion

To the best of our knowledge, this is the first study to explore the expression pattern and mechanism of MPS-1 in endometrioma development. We found that the expression of MPS-1 was significantly higher in endometrioma than in either eutopic endometrium from endometriosis patients or control endometrium. MPS-1 was overexpressed in the serum of patients with endometrioma. MPS-1 downregulation inhibited EcESCs proliferation, migration, and invasion as well as promoted the arrest of the cell cycle in the G0/G1 phase and apoptosis. Furthermore, MPS-1 downregulation suppressed the NF-κB signaling pathway. Our results suggest that MPS-1 plays a role in endometrioma progression, which may contribute to explore the pathogenesis of endometrioma.

MPS-1 is secreted from hyperplastic tissues and can be detected in the serum [23]. MPS-1 is elevated in numerous types of cancer, including breast cancer, lung cancer, and head and neck squamous cell carcinoma [10]. Furthermore, higher levels of MPS-1 are associated with advanced stages of cancer, poorer prognoses, and recurrence in cancer [24]. MPS-1 is overexpressed even in benign diseases that remain in proliferative or active stages, such as rheumatoid arthritis [19]. Although endometriosis is a benign gynecological disease, the ectopic endometrium can develop into endometrioma through several biological processes that include adhesion, proliferation, invasion, and angiogenesis, indicating that endometriotic lesions are in a state of continuous and dynamic progress. Our results show that the expression of MPS-1 is elevated in both endometrioma and in the serum of endometriosis patients compared to non-endometriosis patients. Notably, the serum concentration of MPS-1 could distinguish endometriosis patients from controls and was positively associated with CA-125 levels. Many studies have dedicated to screening endometriosis biomarkers as the golden criteria for endometriosis diagnosis are invasive. Mihalyi et al. generated a panel that consists of six biomarkers (IL-6, IL-8, TNF-α, hsCRP, CA-125, and CA-199) to help diagnose minimal and mild endometriosis [25]. Vodolazkaia et al. combined Annexin-V, vascular endothelial growth factor (VEGF), CA-125, slCAM-1, and glycodelin to diagnose endometriosis that cannot be detected by ultrasonography [26]. According to our data, the presence of MPS-1 in serum may be an indicator of endometriosis. However, whether there exists the possibility to generate diagnostic models combining MPS-1 with other biomarkers warrants further investigation using a larger sample size.

Ribosomal proteins (RPs) are major constituents of the ribosome that control total protein synthesis. RPs not only take part in the translational machinery by serving as scaffold structures, but they also participate in extra-ribosomal functions by interacting with other proteins or RNAs [27]. It has been reported that MPS-1 could regulate gastric cancer cell metastasis through integrin β4 [28]. Interestingly, MPS-1 exerts contrasting functions in different diseases. MPS-1 has been shown to promote colorectal cancer progression by activating JNK/c-Jun signaling [8]. In contrast, overexpression of MPS-1 in multiple myeloma inhibits cell growth in vitro and in vivo by reducing the expression of fibroblast growth factor and inactivating ERK MAPK signaling [29]. The different cell types and varying regulatory mechanisms may account for the different roles of MPS-1 in diseases. Our results show that MPS-1 can promote EcESCs proliferation, migration, invasion, and anti-apoptosis via the NF-κB signaling pathway, which may contribute to the formation, progression, and elimination of ectopic lesions. NF-κB has been reported to be overexpressed in endometriosis, including ectopic lesions, the eutopic endometrium, endometriotic stromal cells, and peritoneal macrophages [15]. Estrogen plays an important role in endometriosis, and there are cell-type-specific interactions between estrogen receptors (ERs) and NF-κB signaling [30]. ERs block the binding of DNA to NF-κB in endometrial stromal cells. However, estrogen enhances NF-κB activity in endometrial epithelial cells. Additionally, the NF-κB pathway can participate in inflammation and the immune response, which are critical to the development of endometriosis [31]. Proinflammatory cytokines, such as IL-1, IL-6, and TNF-α, can activate NF-κB, which induces inflammatory cell infiltration and increases the secretion of cytokines, stimulating the NF-κB signaling pathway and forming a positive feedback loop in endometriosis [31–34]. Subsequently, active NF-κB promotes the transcription of intercellular adhesion molecule, matrix metalloproteinase and vascular endothelial growth factor (VEGF) that are associated with proliferation, adhesion, invasive and angiogenesis processes by binding to the κB sequence on the promoters [15]. VCAM-1 is an important downstream effector of the NF-κB signaling pathway, contributing to the progression of endometriosis. VCAM-1 downregulation can inhibit endometriotic cyst stromal cell proliferation, migration, and invasion mediated by transforming growth factor beta 1 (TGF-β1) [20]. In addition, soluble vascular adhesion molecule-1 (sVCAM-1) in serum is considered a promising biomarker for diagnosing endometriosis [19]. Although our results show that NF-κB signaling is involved in the regulation of MPS-1 in endometriosis, the specific regulatory mechanism between MPS-1 and NF-κB has not been explored. RPS3, a ribosomal protein with a zinc motif can bind to NF-κB to enhance the transcriptional activation of target genes by stabilizing the NF-κB-DNA complex [35]. Hence, we speculated that MPS-1 may regulate NF-κB in a similar manner. The intricate regulatory mechanisms between MPS-1 and NF-κB are worthy of further exploring.

It is important to elucidate the mechanisms responsible for the overexpression of MPS-1 in endometrioma and the regulation of MPS-1 under normal conditions. Sun et al. found that the transcription of MPS-1 is repressed by wild-type p53, while mutated p53 did not have an inhibitory effect on MPS-1 [7]. It is known that the expression of p53 in endometriotic tissues is lower than that in the eutopic endometrium of endometriosis or normal endometrium [36]. In addition, the p53 codon 72 gene polymorphism is associated with endometriosis susceptibility, especially in Latin American and Asian populations [37]. Therefore, the overexpression of MPS-1 may result from both decreased expression and increased mutants of p53 in endometriosis. Furthermore, MPS-1 is a growth factor-induced gene that is stimulated by TGF β-1, serum, and cAMP analogs [38]. It is well known that the expression of TGF β-1 is elevated in the ectopic endometrium, peritoneal fluid, and serum of patients with endometriosis [32, 39]. Whether overexpressed TGF β-1 plays a role in the increase of MPS-1 levels requires further investigation.

Looking back on our research, there are some limitations. We only included endometrioma without other types of endometriosis, whether the differential expression pattern or mechanism of MPS-1 exists in deep infiltrating endometriosis or peritoneal lesions remains unclear. To better elucidate the role of MPS-1 in endometriosis, we will collect different types of endometriotic lesions and lesions in different phases in the future.

## Conclusion

In summary, our results reveal the high expression of MPS-1 in endometriosis and that MPS-1 can regulate multiple vital biological processes, including cell proliferation, motility, invasion, apoptosis, and cycle, via the NF-κB signaling pathway. We believe that our study will contribute to further understanding the complex pathogenesis of endometriosis and clinical transforming prospects.

## Supplementary Information

**Additional file 1: Supplementary Table. **Clinical related information of patients.

**Additional file 2: Supplementary Figure. **Characterization image of EcESCs.Identification of primary ectopic endometrial stromal cells with vimentin (Green) and cytokeratin-7 (Red). The fluorescence was observed under the laser scanning confocal microscope at magnification 100X or 600 X.

## Data Availability

All data generated or analyzed during this study are included in this published article and its supplementary information files.

## Abbreviations

MPS-1: Metallopanstimulin-1
VCAM-1: Vascular cell adhesion molecule-1
EcESCs: Ectopic endometrial stromal cells
qRT-PCR: Quantitative real time polymerase chain reaction
ELISA: Enzyme-linked immunosorbent assay
siRNA: Small interfering RNA
NC: Negative control
RPs: Ribosomal proteins
ERs: Estrogen receptors
TGF-β1: Transforming growth factor beta 1
sVCAM-1: Soluble vascular adhesion molecule-1
