# Supplementary material for: Overexpressed MPS-1 contributes to endometrioma development through the NF-κB signaling pathway
Source: Reprod Biol Endocrinol. 2021 Jul 15;19:111. doi: 10.1186/s12958-021-00796-z (PMC8281640; doi:10.1186/s12958-021-00796-z)
Supplement: Supplementary file 1 — Additional file 1: Supplementary Table. Clinical related information of patients. [file 12958_2021_796_MOESM1_ESM.docx]

**Supplementary Table. Clinical related information of patients**

|  | Control patients | Endometriosis patients  (rAFS III-IV) |
| --- | --- | --- |
| Number | 20 | 20 |
| Age (years) | 32.3 ± 1.5 | 32.3 ± 1.0 |
| leiomyoma | 14 | 7 |
| uterus septum | 6 | 3 |
| Infertility | 3 | 6 |
| Maximum diameter (cm) | 0 | 5.9 ± 0.5 |
|  |  |  |
