# Supplementary figures and images for: Overexpressed MPS-1 contributes to endometrioma development through the NF-κB signaling pathway
Source: Reprod Biol Endocrinol. 2021 Jul 15;19:111. doi: 10.1186/s12958-021-00796-z (PMC8281640; doi:10.1186/s12958-021-00796-z)

**DAPI**

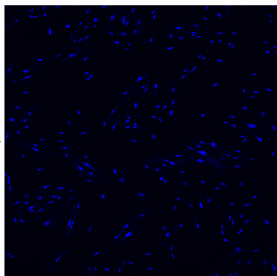

**Vimentin**

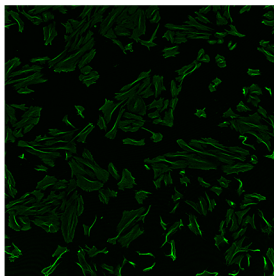

**Cytokeratin-7**

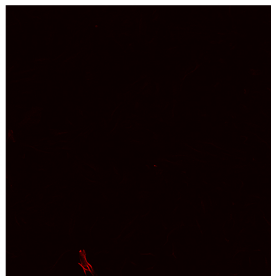

**DAPI**

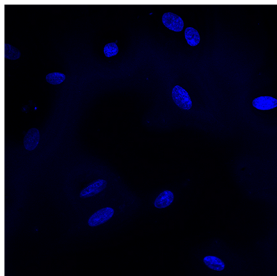

**Vimentin**

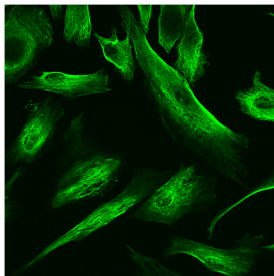

**Merge**

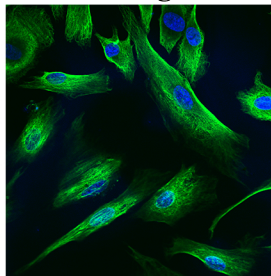

Supplement: Supplementary file 2 — Additional file 2: Supplementary Figure. Characterization image of EcESCs.Identification of primary ectopic endometrial stromal cells with vimentin (Green) and cytokeratin-7 (Red). The fluorescence was observed under the laser scanning confocal microscope at magnification 100X or 600 X. [file 12958_2021_796_MOESM2_ESM.pdf]
